# MicroRNA-196a links human body fat distribution to adipose tissue extracellular matrix composition

**DOI:** 10.1016/j.ebiom.2019.05.047

**Published:** 2019-05-28

**Authors:** Catriona Hilton, Matt J. Neville, Laura B.L. Wittemans, Marijana Todorcevic, Katherine E. Pinnick, Sara L. Pulit, Jian'an Luan, Agné Kulyté, Ingrid Dahlman, Nicholas J. Wareham, Luca A. Lotta, Peter Arner, Cecilia M. Lindgren, Claudia Langenberg, Fredrik Karpe

**Affiliations:** a Oxford Centre for Diabetes, Endocrinology and Metabolism, Radcliffe Department of Medicine, University of Oxford, Churchill Hospital, Oxford OX3 7LE, UK; b NIHR Oxford Biomedical Research Centre, OUH Trust, Oxford OX3 7LE, UK; c Big Data Institute, University of Oxford, Oxford OX3 7FZ, UK; d Wellcome Trust Centre for Human Genetics, Oxford University, Oxford OX3 7BN, UK; e Department of Genetics, University Medical Center Utrecht, Utrecht, the Netherlands; f Medical Research Council Epidemiology Unit, University of Cambridge, Cambridge CB2 0QQ, UK; g Department of Medicine (H7), Karolinska Institutet at Karolinska University Hospital - Huddinge, 141 86 Stockholm, Sweden

**Keywords:** Abdominal, Adipocyte, Body fat distribution, Gluteal, Human adipose tissue, MicroRNA

## Abstract

**Background:**

Abdominal fat mass is associated with metabolic risk whilst gluteal femoral fat is paradoxically protective. MicroRNAs are known to be necessary for adipose tissue formation and function but their role in regulating human fat distribution remains largely unexplored.

**Methods:**

An initial microarray screen of abdominal subcutaneous and gluteal adipose tissue, with validatory qPCR, identified microRNA-196a as being strongly differentially expressed between gluteal and abdominal subcutaneous adipose tissue.

**Findings:**

We found that rs11614913, a SNP within pre-miR-196a-2 at the *HOXC* locus, is an eQTL for miR-196a expression in abdominal subcutaneous adipose tissue (ASAT). Observations in large cohorts showed that rs11614913 increased waist-to-hip ratio, which was driven specifically by an expansion in ASAT. In further experiments, rs11614913 was associated with adipocyte size. Functional studies and transcriptomic profiling of miR-196a knock-down pre-adipocytes revealed a role for miR-196a in regulating pre-adipocyte proliferation and extracellular matrix pathways.

**Interpretation:**

These data identify a role for miR-196a in regulating human body fat distribution.

**Fund:**

This work was supported by the Medical Research Council and Novo Nordisk UK Research Foundation (G1001959) and Swedish Research Council. We acknowledge the OBB-NIHR Oxford Biomedical Research Centre and the British Heart Foundation (BHF) (RG/17/1/32663). Work performed at the MRC Epidemiology Unit was funded by the United Kingdom's Medical Research Council through grants MC_UU_12015/1, MC_PC_13046, MC_PC_13048 and MR/L00002/1.

**Research in context**

**Evidence before this study**

Body fat distribution is a strong predictor of metabolic health, independent of total adiposity. The role of microRNAs is regulating body fat distribution has not been well defined.

**Added value of this study**

We demonstrate that miR-196a shows strong adipose depot-specific expression patterns. A single nucleotide polymorphism in pre-miR-196a-2 influences the expression of miR-196a in abdominal subcutaneous adipose tissue and is associated with waist-hip ratio. Furthermore, functional studies demonstrate that miR-196a regulates pre-adipocyte proliferation and extracellular matrix composition.

**Implications of all the available evidence**

Our study identifies a role for miR-196a in regulating human body fat distribution. MicroRNAs hold potential as therapeutic targets and biomarkers.

Alt-text: Unlabelled Box

## 1 Introduction

The metabolic consequences of adiposity are dictated not only by absolute adipose tissue (AT) mass but also by its distribution [[1], [2], [3], [4], [5]], to the extent that waist-to-hip ratio (WHR) is a stronger predictor of myocardial infarction than body mass index (BMI) [6]. Abdominal subcutaneous, visceral and gluteal femoral AT are structurally [7] and functionally [[8], [9], [10]] distinct. WHR has heritability estimates ranging from 40%–70% [11,12]. To date, the precise genetic mechanisms determining body fat distribution are poorly defined and the top SNPs within associated loci identified in genome-wide association studies explain only a small proportion of the phenotypic variance [13,14]. Other biological factors, epigenetic amongst them and including tissue-specific microRNAs (miRNA) [15], could explain an additional proportion of phenotypic variation.

## 2 Materials and methods

### 2.1 Human subject cohorts

#### 2.1.1 Oxford Biobank (OBB)

The OBB is a cohort of apparently healthy 30–50-year old men and women from Oxfordshire [16]. All volunteers in the OBB have undergone a comprehensive anthropomorphic and metabolic characterisation. ASAT biopsies were taken from the abdominal wall 2 in. lateral to the umbilicus and GSAT biopsies were taken from the upper outer quadrant of the buttock. Biopsies were obtained under local anaesthesia (1% lignocaine) using a 12-gauge needle aspiration technique. Whole-body DXA scans were performed using a Lunar iDXA (GE Healthcare) and images were processed using enCORE v14.1 software (GE Healthcare). The android region is defined by the iliac crest at the lower boundary and the upper boundary is calculated as 20% of the distance between the neck and the iliac crest. The gynoid region includes the upper thighs and hips but does not overlap with the umbilicus. It is twice the height of the android region with the upper boundary located below the iliac crest by 1.5 times the height of the android region. VAT is calculated by the encore v14.1 software using a predefined algorithm. Android subcutaneous AT is calculated as total android AT - VAT. Ethical approval for studies involving OBB participants was granted by Oxfordshire Clinical Research Ethics Committee (08/H0606/107) and all study participants have given written informed consent.

#### 2.1.2 Paired ASAT and VAT biopsies

Paired whole VAT and ASAT surgical biopsies were obtained from sixteen Caucasian women (BMI 43∙1 (36∙9–47∙6) kg/m^2^, age 44∙5 (42∙8–48∙5) years) who were undergoing bariatric surgery as previously described [17].

#### 2.1.3 GIANT consortium

The GIANT consortium has access to anthropometric and genotyping data for nearly 250,000 individuals. Shungin *et al* [14] conducted a meta-analysis of waist and hip circumference measurement associations, adjusted for age, BMI and study specific covariates, in individuals of European ancestry using data from 57 GWAS studies. The *p*-values were corrected using genomic control at the individual study level and again after meta-analysis. Manhattan plots for the meta-analysis are available here [14].

#### 2.1.4 UK Biobank

The UK Biobank includes anthropometric, biochemical and genetic data from >500,000 men and women recruited between 2006 and 2010 (REC 11/NW/0382). Further details are available elsewhere [18].

#### 2.1.5 EPIC-Norfolk

The EPIC-Norfolk cohort includes 25,000 men and women who were aged between 40 and 79 at recruitment and who underwent DXA imaging for body composition at the 20 year follow up assessment.

#### 2.1.6 Fenland cohort

The Fenland Study is a population-based cohort study of the interaction between genetics and environment in the development of diabetes and obesity. The cohort contains a Cambridgeshire-based population of men and women without diabetes born between 1950 and 1975. Participants undergo detailed anthropometric and metabolic characterisation along with genotyping and DXA determination (GE Lunar iDXA) of body composition as recently described [5].

#### 2.1.7 Karolinska Institute cohorts

Genotyping and phenotypic data on fat cell size in ASAT was obtained as described previously [19].

### 2.2 Primary cell cultures and region-specific human immortalised pre-adipocyte cell lines

Primary pre-adipocytes were obtained by collagenase digestion of minced whole AT biopsies. Volunteers for AT biopsy were recruited from the OBB and were not included in other datasets involving OBB subjects. For determination of miR-196a in fractionated AT the floating adipocyte layer and stromal-vascular pellet were isolated after collagenase digestion and fractionation (*n* = 1). The _im_APAD and _im_GPAD cell lines have been described previously [20]. For cell culture experiments pre-adipocytes were cultured in growth medium (Dulbecco's Eagle's Medium Nutrient Mixture (DMEM)/F12 HAM (*v*/v, 1:1), 10% foetal calf serum (FCS) (Invitrogen, Paisley, UK), 0.25 ng/ml fibroblast growth factor (FGF), 100 U/ ml penicillin and 0.1 mg/ ml streptomycin). All cell cultures were incubated at 37 °C with 5% CO_2_. To differentiate pre-adipocytes, confluent cells were cultured for 14 days with differentiation media (DMEM/F12 HAM (1:1) containing 2 mM glutamine, 17 μM pantothenate, 100 nM human insulin, 1 nM triiodo-L-thyronine (T3), 33 μM biotin, 10 μg/ ml transferrin and 1 μM dexamethasone, with the addition of 3-Isobutyl-1-methylxanthine (IBMX) (250 μM) and troglitazone (4 μM) for the first 4 days).

Human Embryonic Kidney 293 (HEK293) cell lines were cultured in growth media consisting of DMEM with 4500 mg/l glucose, 10% FCS, 2 mM glutamine, 100 U/ ml penicillin and 0.1 mg/ ml streptomycin.

### 2.3 Generation and culture of stable miR-196aKD cell lines

MISSION custom hsa-miR-196a-5p inhibitor [21] and ath-miR-416 negative control cloned into the TRC2-pLKO-puro vector were purchased from Sigma-Aldrich (USA) in the form of DNA plasmids. Plasmids were amplified in MAX Efficiency DH5α Competent *E. coli* (Invitrogen, UK). Amplified plasmids were isolated using the Midiprep plasmid kit (Qiagen, UK).

Lentiviral particles were produced by co-transfection of HEK293 cells with the MISSION hsa-mir-196a-5p inhibitor or ath-miR-416 negative control vector along with packaging vectors (MISSION packaging mix, Sigma-Aldrich, UK) using Fugene 6 (Promega, UK). To generate stable pre-adipocyte cell lines, _im_APAD and _im_GPAD cell line pre-adipocytes at passage 8 were plated in T25 flasks at a density of 1∙2x10^5^cells/flask in complete growth media. Cells were transduced by culturing in complete growth media with the addition of lentiviral particles and hexadimethrine bromide at a final concentration of 8 μg/ml. Pre-adipocyte cell lines were cultured in the presence of 2 μg/ml puromycin during the proliferative phase but not after the addition of differentiation media. The stable cell lines generated are referred to as _im_APAD mir-196aKD, _im_APAD-Con, _im_GPAD mir-196aKD and _im_GPAD-Con. Intracellular lipid levels were quantified using the AdipoRed assay reagent (Lonza) and a CytoFluor Multi-Well Plate Reader series 4000 (PerSeptive Biosystems). To calculate doubling time, pre-adipocytes were seeded at equal density in T75 flasks and were trypsinised and triple counted every 72 h. Doubling time was calculated using the formula:

Doubling time = t2-t1 ((log [2]/log(q2/q1)).

where t = time (days) and q = cell number.

## 3 Method details

### 3.1 RNA extraction and quantification

RNA was isolated from Tri-reagent. For microarray experiments RNA was purified using MirVana Columns (Life Technologies). For other experiments RNA was purified using a standard Tri-reagent protocol. cDNA was synthesised using the miScript kit (Qiagen). For mRNA quantification qPCR was performed using Taqman Assays-on-Demand (Applied Biosystems) and Kapa Probe Fast Mastermix (Kapa Biosystems) in a 6 μl final volume. For microRNA quantification Qiagen miScript primer assays were used with the QuantiTect SYBR Green PCR Kit (Qiagen, UK) in an 8 μl reaction. Gene expression was quantified using the ΔΔCT method [22]: mRNA was quantified relative to the average expression of peptidylprolyl isomerase A (*PPIA*) and phosphoglycerate kinase 1 (*PGK1*); miRNA and pre-miRNA abundance was quantified relative to the average of miR-103, miR-24 and miR-331 [23]. Readings of fluorescence detected during the qPCR reaction on the ABI Prism 7900HT were analysed using SDSv2.3 software (Life Technologies, UK).

### 3.2 Genotyping

Genomic DNA was extracted from whole blood by LGC Genomics (Hoddesdon, UK). Genotyping in the OBB cohort was performed on 10 ng of DNA using KASP On Demand custom designed and validated primers for rs11614913 (LGC Genomics, Hoddesdon, UK). Allelic discrimination performed was on the ABI Prism 7900 HT Real-Time PCR System (Life Technologies, UK) using SDSv2.3 software (Life Technologies, UK).

### 3.3 Western blotting

HEK293 cell lines transduced with miR-196a KD or control vector were cultured in 6-well plates and then lysed by the addition of 150 μl of lysis buffer (50 mM Tris pH 8.0, 250 mM NaCl, 5 mM EDTA, 0.5% and Igepal CA-630). Normalised protein samples were resolved on a Criterion Stain-Free precast gel (Bio-Rad, UK). Protein transfer onto a polyvinylidene fluoride (PVDF) membrane was performed using the Trans-Blot Turbo system (Bio-Rad, UK). The membrane was visualised with the ChemiDoc MP system (Bio-Rad, UK) to quantify total protein in each lane. The membrane was probed with HOXC8 rabbit polyclonal antibody (ab86236 Abcam, RRID: AB_1925078) at a 1:1000 and 1:1000 goat anti-rabbit IgG horseradish peroxidase (HRP)-conjugated secondary antibodies (DAKO). An enhanced chemiluminescence (ECL) reaction was performed using Clarity ECL substrate (Bio-Rad, UK) and was visualised using both the ChemiDoc MP and imaging film. HOXC8 was quantified and normalised to total protein using Image Lab software (Bio-Rad, UK).

### 3.4 Microarray of whole ASAT and GSAT

ASAT biopsies from 15 men (8 lean (BMI < 25) and 7 overweight (BMI > 25)) and paired GSAT biopsies from 8 of the men (5 lean and 3 overweight) were hybridized to individual Agilent Human microRNA Microarrays v2.0 (Agilent Technologies, Santa Clara, USA). The DNA microarray scanner (Agilent G2565BA) was used to scan hybridized microarrays and features were extracted using the Agilent Feature Extraction image analysis tool (version 9.5.1) with default protocols and settings. Data were background corrected and quantile normalised in R [24] using the Bioconductor AgiMicroRna package [25]. Differential expression analysis was performed in Genesifter (Geospiza, Seattle, USA) with an uncorrected *p*-value of 0∙05 indicating significance. A minimum fold-change of 20% was selected to identify biologically important differences in expression.

### 3.5 Microarray analysis of miR-196aKO cell lines

Six replicates of passage 10 _im_APAD mir-196aKD, _im_APAD-Con, _im_GPAD mir-196aKD and _im_GPAD-Con pre-adipocytes were harvested in Tri-reagent. Cells were harvested on day 3 after plating and were sub-confluent. RNA was isolated using the mirVana miRNA Isolation Kit (Life Technologies). RNA concentration was assessed using the Nanodrop ND-1000 spectrophotometer and by RiboGreen® assay and samples were normalised to give 100 ng RNA in a total volume of 11 μl. RNA quality was verified using the Agilent TapeStation (Agilent, Santa Clara, US). RNA samples were reverse transcribed using the Illumina TotalPrep-96 RNA Amplification Kit and then converted into labelled cDNA using the Illumina Whole-Genome Gene Expression Direct Hybridisation Assay. The labelled ss-cDNA was then hybridized to two HumanGene2.1 ST-16 Array Plates. The array was washed, stained and scanned using the Affymetrix GeneTitan platform. Raw data were Robust multi-array (RMA) normalised and checked for quality. Differential expression analysis was performed in R [24].

Gene ontology (GO) enrichment analysis was performed in Database for Annotation, Visualization, and Integrated Discovery (DAVID) (http://www.david.niaid.nih.gov/ [26,27]) with the gene list from the Affymetrix HumanGene2.1 ST-16 array as background. EASE score was used to calculate enrichment for GO classifications. The default functional annotation cluster function within DAVID was used to cluster redundant or closely related GO terms together. The enrichment score for each cluster is the geometric mean of the Bonferroni-corrected *p*-values for the cluster. Transcripts were excluded from analysis if expression was below background intensity in both conditions being compared.

### 3.6 Credible sets analysis

To generate credible sets of likely-causal variants at the *HOXC* locus, we first identified all of the independent signals in the locus using approximate conditional testing in Genome-wide Complex Trait Analysis (GCTA) [28] using the GIANT summary-level data in European-ancestry samples only. Genotyping data from the PIVUS cohort (*N* = 949), included in the GIANT analysis, was used as the reference dataset in the GCTA analysis. Once we had identified the independent signals contained in the locus, we performed conditional testing (also in GCTA) to generate summary-level data for each signal. For example, to generate summary-level data for independent signal A (index SNP rs10783615), we conditioned on independent signals B (index SNP rs1443512) and C (index SNP rs2071449) and then stored the resulting summary-level statistics. We repeated this process for signals B and C. We used the index SNPs as reported in the initial genome-wide association study of WHR performed in GIANT [14]. Finally, once we had generated summary-level data for all three signals, we used a Bayesian model as previously implemented in a genome-wide association study of type 2 diabetes to determine the credible sets of likely-causal SNPs in the region (for each of the sets of summary-level data). The analysis results in a posterior likelihood for each SNP that the SNP is the causal variant in the region.

We performed an identical analysis using the UK Biobank data. We used the individual level genotypes from the UK Biobank as a reference for the GCTA analyses, and used the same three SNPs as index SNPs for conditional testing.

### 3.7 Statistical analyses

Statistical analyses were performed in SPSS 20.0 or 22.0. All *p*-values are two-tailed.

### 3.8 Data sharing

Supplementary data for the microarray analysis of microRNAs in ASAT tissue from 15 men, with paired gluteal adipose tissue from 8 of the men (DOI: 10.17632/krgbnvxc5v.1) and for miR-196a KD in immortalised abdominal and gluteal pre-adipocytes (DOI: 10.17632/m4xtmzmx39.1) are available at Mendeley (https://data.mendeley.com).

## 4 Results

### 4.1 MicroRNA-196a displays adipose tissue depot-specific expression patterns

**Fig. 1:**
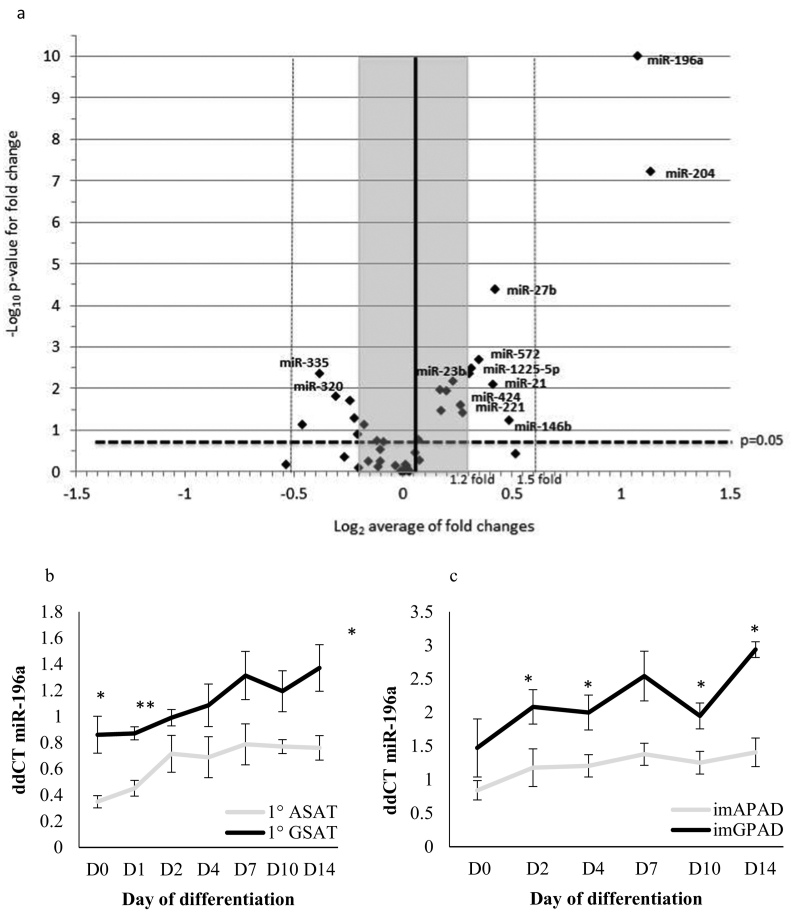
a. Volcano plot showing qPCR validation data of selected differentially expressed miRNAs between gluteal and abdominal subcutaneous adipose tissue (*n* = 40 paired biopsies). Positive log_2_ fold changes indicate higher expression in gluteal adipose tissue. Those that retained significance are indicated with name and their data is presented in Table S2. b. miR-196a expression in *in vitro* differentiated primary pre-adipocytes derived from ASAT and GSAT (*n* = 6; mean ± SE; *p < 0∙05, **p < 0∙005, paired *t*-test). c. miR-196a expression in *in vitro* differentiated _im_APAD and _im_GPAD cell lines derived from ASAT and GSAT respectively (n = 6; mean ± SE; * p < 0∙05, paired *t*-test).

To assess the extent to which miRNAs may contribute to body fat distribution, we performed microarray miRNA profiling of abdominal subcutaneous adipose tissue (ASAT) from 15 men recruited from the Oxford Biobank (OBB) (mean age 43∙8 years, 35–54 years; 8 lean (BMI < 25 kg/m^2^) and 7 overweight (BMI > 25 kg/m^2^)) together with paired gluteal subcutaneous adipose tissue (GSAT) from 4 lean and 4 overweight men. Of the 161 miRNAs included in the array and expressed in AT, 32 reached the significance threshold (*p* = 0∙05) for differential expression with a fold change of at least 20% (Table S1). Validation qPCR was performed on paired ASAT and GSAT biopsies from an expanded panel of 20 men and 20 women from the OBB and including both lean and overweight BMI categories (Table S2). Twelve of the original miRNAs remained significant after validation (Table S3, Fig. 1a). Two miRNAs, miR-196a and miR-204, were the most differentially expressed miRNAs between ASAT and GSAT, both with 2∙3-fold higher expression in GSAT (miR-196a: *p* = 9∙6 × 10^−11^; miR-204: *p* = 6∙2 × 10^−8^, paired *t*-test). MiR-196a is of particular interest because it is found within the *HOXC13* locus, which has been linked to WHR adjusted for BMI in large-scale genome-wide association studies [14,29]. Several studies have demonstrated that miR-196a is necessary for embryonic patterning [[30], [31], [32]]. Studies of miR-196a expression in other species and non-adipose tissues have shown increasing expression moving distally along the anterio-posterior axis [31,[33], [34], [35]]. Further, miR-196a appears functional in human adipocytes: Mori et al. proposed that miR-196a regulates brown adipogenesis of white AT lineage cells by targeting *HOXC8,* which in turn regulates the adipogenic signal *CEBPβ* [36]. In the expanded panel of 40 individuals miR-196a was strongly different between ASAT and GSAT but was not influenced by obesity (ASAT: *p* = 0∙18; GSAT: p = 0∙82, independent samples *t*-test). We therefore hypothesised that miR-196a could have a functional role in determining body fat distribution.

Further dissection of miR-196a expression in AT using the expanded follow-up panel of 40 individuals (Table S2) demonstrated it was not influenced by sex (ASAT: p = 0∙55; GSAT: p = 0∙069, independent samples *t*-test). The depot-specific expression pattern of miR-196a was seen in both men and women (Table S3).

We next performed *in vitro* differentiation time-courses of both primary pre-adipocytes and immortalised human pre-adipocytes derived from ASAT and GSAT (termed _im_APAD and _im_GPAD respectively [20]). Expression of adipogenic transcription factors (*PPARG2, CEBPA, CEBPB* and, *CEBPD)* and markers of terminal adipocyte differentiation (*PLIN1, INSR, ADIPOQ, CD36* and *LPL*) was similar for both primary and immortalised differentiated pre-adipocytes derived from both depots, although cells of GSAT origin accumulated significantly more intracellular lipid (data shown in [20]). The miR-196a fat depot-specific expression pattern was maintained *in vitro* throughout differentiation in both the primary adipocyte culture and in the immortalised cell lines (Figs. 1b-c), suggesting that its expression pattern may be intrinsic to the location-specific pre-adipocytes and not a function of the *in vivo* environment. MiR-196a was up-regulated during adipocyte differentiation (Figs. 1b-c). Consistent with this finding, miR-196a expression was 7∙1-fold higher in the mature adipocyte fraction compared with the pre-adipocyte containing stromo-vascular fraction of AT. Within an independent panel of 16 within-person paired samples obtained from women undergoing bariatric surgery [17] expression of miR-196a was extremely low in visceral AT (VAT) (25-fold lower, *p* = 5∙0 × 10^−6^; paired *t*-test) compared with ASAT, confirming the depot-specific expression pattern. Additionally, miR-196a is highly expressed in human AT compared to other tissues: a previous analysis of high-throughput sequencing data from 131 individuals from the MuTHER cohort [37] revealed that miR-196a expression was significantly higher in ASAT than other human tissues except for 2 other mesenchymal tissues, muscle and kidney.

The homeobox miR-196a target mRNAs *HOXA5* [38]*, HOXB8* [33,39] and *HOXC8* [36,39,40] displayed reciprocal expression with miR-196a in the expanded panel of 40 individuals (*HOXA5*: 5-fold lower in GSAT *vs* ASAT, *p* < 0∙001; *HOXB8*: 3-fold lower in GSAT, *p* = 0∙16; *HOXC8*: 2-fold lower in GSAT, p < 0∙001). These patterns were maintained during *in vitro* differentiation of primary pre-adipocytes and the immortalised pre-adipocyte cell lines (Fig. S1).

There are three paralogues of the mir-196 gene located on different chromosomes: mir-196a-1 and 2 transcribe the same mature miRNA which cannot be distinguished by qPCR, but are derived from unique precursor sequences; miR-196b differs from mir-196a-1 and 2 by one nucleotide. Pre-miR-196a-1 was not detected by qPCR in either AT depot in the expanded panel (Table S2), whilst pre-miR-196a-2 showed depot-specific expression mirroring mature miR-196a (*n* = 40, 2.4-fold higher in GSAT than ASAT, *p* = .006, Wilcoxon signed rank test), which shows that miR-196a-2 is the predominant paralogue in human AT.

### 4.2 A SNP in pre-mir-196a-2, rs11614913, is associated with human body fat distribution

Previous genome-wide efforts have used conditional association testing to identify three independent signals for WHR adjusted for BMI harboured within the *HOXC* locus (defined for simplicity as signals A, B and C [14]). The sentinel SNP defining the C signal (rs2071449), is within the LD block also containing the SNP rs11614913 which falls within the pre-mir-196a gene region (linkage disequilibrium (r^2^) = 0.59 with rs2071449 indicating moderate linkage disequilibrium). To investigate whether rs11614913 has any functional consequences and is linked to the associations observed for this region we first confirmed an association between rs11614913 and body fat distribution. Meta-analysis data from the GIANT consortium [14] performed in 150,738 individuals revealed an association between WHR (adjusted for BMI and age) and the minor allele (T) of rs11614913 (*n* = 141,414, beta (se) = 0∙03 (0∙004), *p* = 6∙9 × 10^−11^) in both men (55,920, beta (se) = 0∙03 (0∙007), *p* = 2∙7 × 10^−5^) and women (*n* = 85,629, beta (se) = 0∙02 (0∙006), *p* = 1∙1 × 10^−7^). This was mediated by an expansion in waist circumference (*n* = 150,738, beta (se) = 0∙03 (0∙004), p = 1∙7 × 10^−11^; men: *n* = 60,340, beta (se) = 0∙03 (0∙007), p = 1∙2 × 10^−6^; women: *n* = 90,592, beta (se) = 0∙03 (0∙005), p = 6∙8 × 10^−8^) rather than an association with hip circumference (*n* = 142,467, beta (se) = 0∙0003 (0∙005), *p* = 0∙95; men: *n* = 56,413, beta (se) = 0∙004 (0∙007), *p* = 0∙53; women: *n* = 86,241, beta (se) = −0∙0016 (0∙006), p = 0∙78). The association between the rs11614913 TT genotype and WHR adjusted for BMI replicates in the UK Biobank sample [18] (*n* = 120,126, beta (se) = 0∙03 (0∙004), p = 1∙4 × 10^−10^; men: n = 56,863, beta (se) = 0∙02 (0∙004), *p* = 3∙8 × 10^−3^; women: *n* = 63,308, beta (se) = 0∙03 (0∙004), p = 1∙04 × 10^−9^). Furthermore, within the OBB population rs11614913 was associated with lean mass of the arms (*n* = 4484, beta (se) = −0∙019 (0∙007), p = 0∙008) following adjustment for age, gender, height squared and first four principal components. We did not observe a significant association between rs11614913 genotype and total lean mass (n = 4484, beta (se) = −0∙010 (0∙007), p = 0∙16) or lean mass of the legs (n = 4484, beta (se) = −0∙0089 (0∙008), p = 0∙28), in keeping with the upper body regionality of the miR-196a AT phenotype.

To further characterise the contribution of this complex region to the observed WHR association, we used GIANT European-ancestry data (*n* = 136,154) and UK Biobank data (*n* = 120,126) to perform conditional testing on each of the three independent *HOXC* loci signals in each respective dataset. Using a Bayesian-refinement approach [41], no single SNP within the locus, including rs11614913, captured the bulk of the posterior probability of being the causal SNP at the locus (Fig. S2). We therefore analysed datasets with better definition of human fat distribution using dual X-ray absorptiometry (DXA, GE Lunar iDXA). The standard output from this gives total android fat mass and calculated visceral fat mass. The ASAT fat mass was calculated by subtracting visceral fat mass from total android fat mass. Analysis of the association between rs11614913 and defined fat depots in a cohort combining the OBB, the UK Biobank, the EPIC-Norfolk and the Fenland cohort (in total *n* = 24,010) showed that rs11614913 was specifically associated with ASAT expansion (ASAT: *n* = 23,945 beta 0∙053 (se) = (0∙0093), *p* = 1∙05 × 10^−8^, Table S4). We therefore investigated whether there was a functional mechanism by which rs11614913 might be causal for AT expansion.

### 4.3 rs11614913 is an eQTL for miR-196a in human adipose tissue

**Fig. 2:**
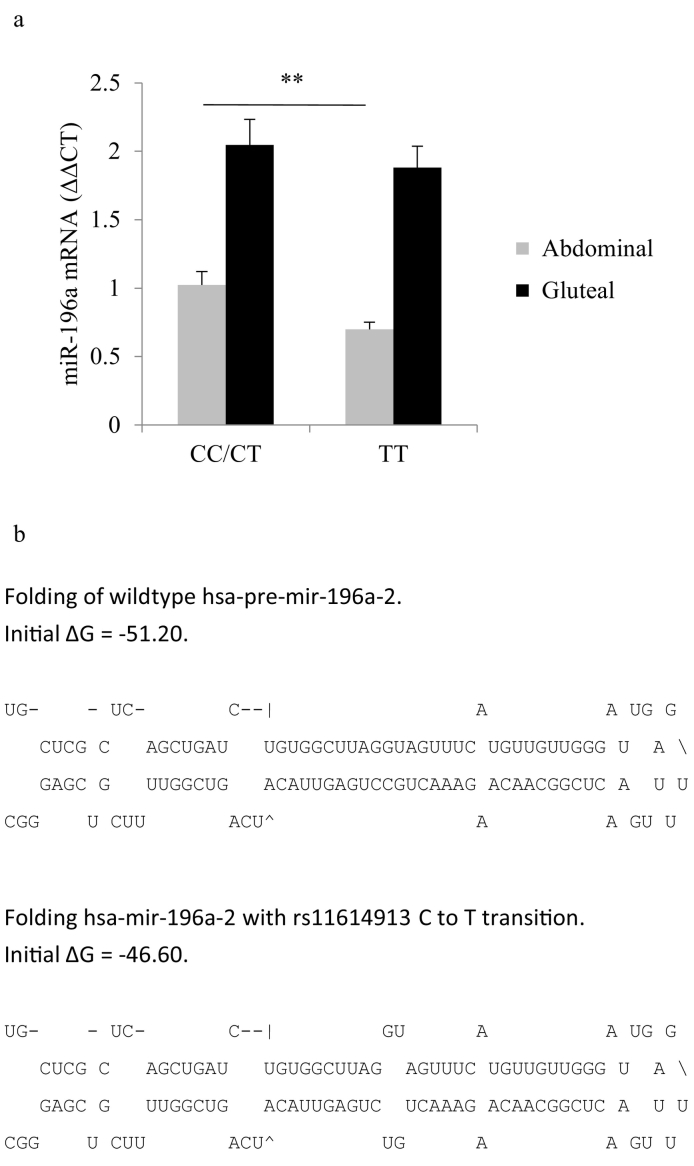
a. miR-196a expression in paired ASAT and GSAT biopsies from age, sex and genotype-balanced group of rs11614913 (*n* = 30 in each group), **p < 0∙005; *t*-test. b. Folding and free energy of binding of miR-196a-2 containing the wild-type allele (top) and minor allele (bottom), as predicted in M-fold.

A separate earlier study including 70 individuals did not identify rs11614913 as an eQTL for miR-196a [42], so to increase the power of the observation of the minor allele (MAF = 0.405) carriers we performed a genotype-balanced recruitment of 90 individuals from the OBB matched for age, sex and BMI for rs11614913 genotypes (*n* = 30 of each genotype CC, CT and TT, *i.e.* selective enrichment of minor allele), and determined miR-196a expression by qPCR in paired ASAT and GSAT biopsies. Expression of miR-196a was lower in ASAT for TT carriers (whole cohort: −32%, mean ddCT (se) TT = 0∙70 (0∙053) CC/CT = 1∙02 (0∙098), *p* = 0∙005, *t*-test; Fig. 2.a; men: −25%, *p* = 0∙011; women: −34%, *p* = 0∙18), consistent with a recessive effect, but not quite statistically significant in GSAT (−8%, mean ddCT (se) TT = 1∙88 (0∙16), CC/CT = 2∙05 (0∙19), p = 0∙061, *t*-test, men: −9%, p = 0∙38; women: −10%, p = 0∙089) which has the higher overall expression. We saw no difference in levels of *HOXA5*, *HOXB8* or *HOXC8* between genotype groups in the panel of 90 individuals (CC/CT *vs* TT; ASAT *HOXA5*: p = 0∙76; GSAT *HOXA5*: 1∙15-fold, p = 0∙24; ASAT *HOXB8*: 0∙81-fold, p = 0∙70; GSAT *HOXB8*: 0∙84-fold, p = 0∙08; ASAT *HOXC8*:0∙94-fold, p = 0∙66; GSAT *HOXC8*: 0∙99-fold, p = 0∙97). However, within the larger MuTHER dataset [43] rs11614913 TT genotype was associated with increased *HOXC8* expression in ASAT (*n* = 856, beta = 0∙043, p = 0∙015). Within the panel of 90 individuals there was no effect of the genotype on pre-miR-196a-2 expression (ASAT: p = 0∙43 Mann-Whitney *U* test; GSAT: p = 0∙17, Mann-Whitney *U* test), suggesting that the effect of the T allele is occurring post-transcriptionally at the level of pre-miRNA processing. The rs11614913 is within the pre-miR-196a-2; *in silico* modelling of pre-miR-196a-2 in M-fold [44] predicted that the rs11614913 C to T transition would increase the free energy of binding (ΔG) of the precursor hairpin from −51∙20 to −46∙60 kCal/mol, destabilising it (Fig. 2b). We therefore predicted that the reduced stability of the minor T allele precursor would reduce the efficiency of cleavage of pre-miR-196a-2.

### 4.4 miR-196a is a negative modulator of proliferation of both abdominal subcutaneous and gluteal preadipocytes

**Fig. 3:**
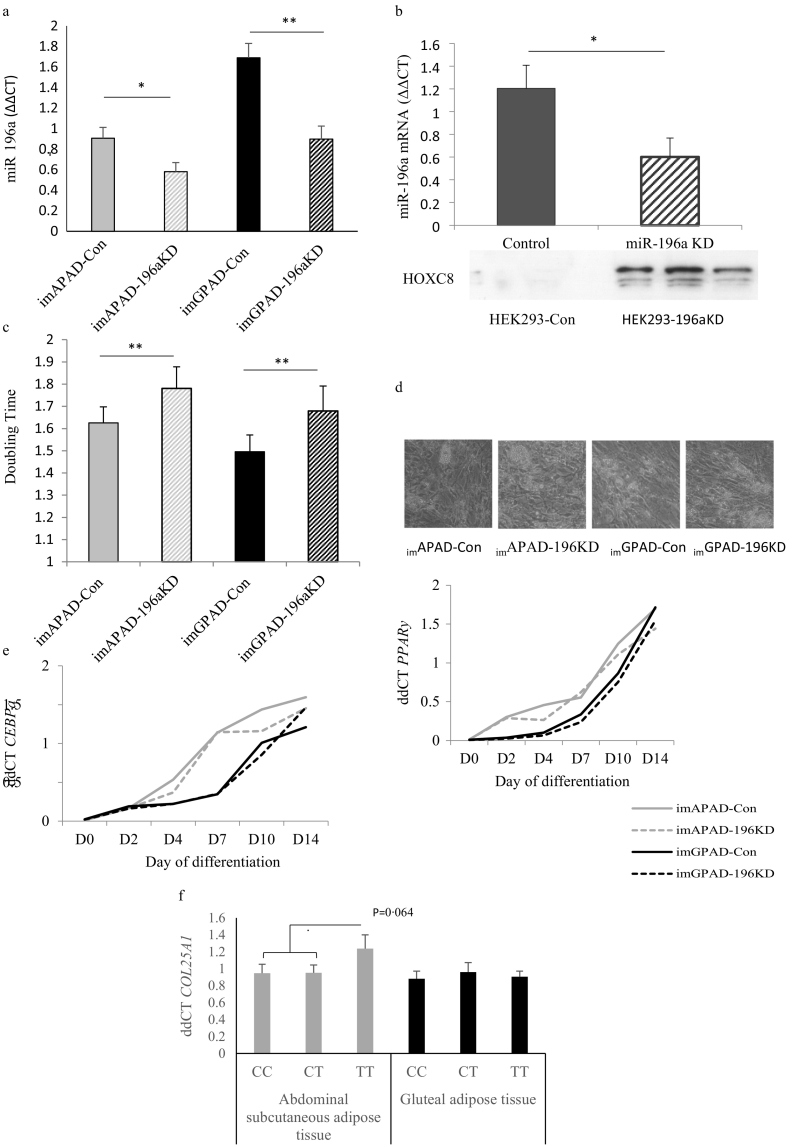
a. Expression of miR-196a in _im_GPAD and _im_APAD cell lines following transduction of miR-196aKD or control vector (*p < 0∙05 **p < 0∙005; paired *t*-test). b. miR-196a and HOXC8 protein in HEK293 cell lines following transduction of miR-196aKD or control vector (*p < 0∙05; paired *t*-test). c. Preadipocyte doubling time of the _im_GPAD and _im_APAD cell lines following transduction of miR-196aKD or control vector, **p < 0∙005. d. Light microscopy (x10 magnification) of the _im_GPAD and _im_APAD cell lines following transduction of miR-196aKD or control vector after 14 days of *in vitro* differentiation. e. mRNA expression of *PPARγ2* and *CEBPα* throughout the 14-day differentiation time-course. f. *COL25A1* expression in paired ASAT and GSAT biopsies from age, sex and genotype-balanced group of rs11614913 (*n* = 30 in each group).

To investigate if miR-196a influences adipogenesis we generated stable miR-196a knock down (KD) cell lines by lentiviral transduction from immortalised pre-adipocyte cell lines (_im_APAD and _im_GPAD), which are derived from ASAT and GSAT respectively [20]. miR-196aKD led to decreased miR-196a expression mirroring the magnitude of difference between rs11614913 TT and CC/CT genotypes (Fig. 3a). As a functional proof-of-concept, the corresponding KD of miR-196a in the HEK293 cell line showed increased protein and gene expression of the established miR-196a target *HOXC8* [36,40] (Fig. 3.b). The miR-196a KD led to a reduced proliferation rate (increased doubling time) of pre-adipocytes of both ASAT and GSAT origin (Fig. 3c; _im_APAD-mir-196aKD *vs*_im_APAD-Con = 9∙5%, *p* = 0∙002; _im_GPAD-mir196aKD *vs*_im_GPAD-Con = 12∙3%, p = 0∙002). There was no difference in adipogenesis of control *vs.* miR-196aKD pre-adipocytes, as determined by microscopy (Fig. 3d), *CEBPa* and *PPARy* expression (Fig. 3e) or cellular lipid (adipoRed) accumulation (_im_APAD mir-196aKD *vs*_im_APAD-Con: *n* = 6, p = 0∙38, paired *t*-test; imGPAD mir-196aKD *vs*_im_GPAD-Con: n = 6, p = 0∙78, paired *t*-test). These data suggest that lower miR-196a expression reduces the tissue adipocyte cellularity *via* adipocyte hypertrophy rather than hyperplasia. Histological examination of whole ASAT biopsies was consistent with this: in ASAT needle biopsies from an independent cohort [19] the mean adipocyte volume in ASAT was 3∙0% greater in male rs11614913 homozygous TT carriers (associated with reduced miR-196a ASAT expression) after adjustment for age and BMI (*n* = 81, beta = 0∙15, p = 0∙019), but not in women (*n* = 232, beta = 0∙018, p = 0∙64).

### 4.5 Transcriptome analysis reveals that miR-196a regulates genes involved in extracellular matrix pathways

To identify AT features that would sustain the observed adipocyte hypertrophy we performed microarray profiling of _im_APAD and _im_GPAD pre-adipocytes transduced with control or miR-196a KD vector. Pre-adipocytes were harvested on day 3 after plating and were sub-confluent. Gene ontology (GO) enrichment analysis of 441 annotated transcripts significantly regulated between _im_GPAD miR-196aKD and _im_GPAD-Con demonstrated a significant enrichment for GO terms related to extracellular matrix (ECM) and vascular development (Benjamini-Hochberg adjusted data) (Table 1), suggesting a role for miR-196a in AT structure and remodelling. The number of annotated transcripts regulated by miR-196aKD in the _im_APAD cell line was lower (Table 1, column 2) but the transcripts with the greatest fold-changes were also enriched for GO terms relating to ECM (Table 1, column 3). We identified *COL25A1,* which is a key ECM component, as strongly up-regulated by miR196aKD in both _im_GPAD and _im_APAD cell lines. This finding was related back to the paired ASAT and GSAT biopsies from the genotype-balanced recruitment of 30 age, sex and BMI-matched individuals which showed 30∙1% higher expression of *COL25A1* in rs11614913 TT carriers (though, possibly due to available sample size, the finding was not quite statistically significant: *p* = .06) indicating that reduced abundance of miR196a may affect matrix expression *in vivo* (Fig. 3f). MiR-196a dysregulation has been observed in scleroderma [45,46] and keloid scarring [47], both pathologies underpinned by ECM abnormalities. Functional studies have shown that miR-196a inhibits production of type I [[45], [46], [47]] and type III collagen [47]. In addition to providing a structural matrix for AT, ECM plays a role in determining the ability of AT to expand [48]. Thus, appropriately configured ECM is necessary for *in vivo* adipogenesis [49], whilst excessive accumulation of ECM components can restrict AT expansion [48].

**Table 1:** Gene ontology terms with significant enrichment for annotated transcripts.

_im_GPAD -196aKD *versus*_im_GPAD-Con		_im_APAD -196aKD *versus*_im_APAD-Con		_im_APAD -196aKD *versus*_im_APAD-Con: based on transcripts with > 50% fold change and significance p < 0∙01	
(*n* = 441 transcripts)		(*n* = 22 transcripts)		(*n* = 47 transcripts)	
Gene ontology terms	Enrichment score	Gene ontology terms	Enrichment score	Gene ontology terms	Enrichment score
Extracellular matrix	9∙14	Inflammation and immune response	2∙92	Extracellular region	3∙26
Vasculature development	6∙98	Interleukin 1	1∙85	Hormone, amidation	2∙00
EGF-like region	6∙48	Positive regulation of macromolecule biosynthetic process	1∙76	Extracellular matrix	1∙83
EGF‑calcium binding	5∙36	Acute inflammatory response	1∙74	Cell motility	1∙42
Cell migration	5∙17	Leucine-rich repeat	1∙67	Negative regulation of transcription	1∙41
Insoluble/membrane fraction	4∙59	Angiogenesis	1∙58		
Polysaccharide and carbohydrate binding	4∙10				
Fibronectin	3∙20				
Positive regulation of transcription	3∙05				
Regulation of apoptosis	2∙53				

## 5 Discussion

The *MIR196A* gene lies within the LD block defined by rs2071449, which is an intronic SNP within *HOXC4* and *C5*, and the sentinel SNP for the WHR adjusted for BMI phenotype association in the region [14]. We showed that rs11614913 may have direct impact on the processing of the pre-mir-196a-2 and functional work identified that homozygote carriers have an altered cellular phenotype. This study is the first to provide a causal mechanism linking to human fat distribution and distinct fat depot expansion at the *HOXC* locus. It should be noted that the populations used in these studies were almost exclusively of European ancestry, and further studies will be required to validate these findings in other populations. Men and women have markedly different body fat distribution. Whilst rs11614913 is associated with increased WHR in both sexes, the association between rs11614913 and adipocyte size was significant in men only. Therefore, *in vitro* miR-196aKD experiments were conducted using immortalised pre-adipocytes from a male donor. Further work is indicated to determine whether there is sexual dimorphism in the actions of miR-196a.

In conclusion, miR-196a, which shows strong AT depot-specific expression, is under genetic influence and exerts a regulatory effect on AT structure and human body fat distribution by affecting the transcriptome for ECM.

Table 1: Gene ontology terms with significant enrichment for annotated transcripts differentially expressed between _im_GPAD -196aKD and _im_GPAD-Con cell lines and between _im_APAD -196aKD and _im_APAD-Con cell lines, after Benjamini-Hochberg correction for multiple testing (*p* < 0∙05). Column 3 refers to annotated transcripts differentially expressed between _im_APAD -196aKD and _im_APAD-Control cell lines with a fold change >50% and an unadjusted significance of p < 0∙01. The enrichment score for each cluster is the geometric mean of the Bonferroni-corrected *p*-values for the cluster.

## Appendix A Supplementary data

Supplementary material Image 1
