# Supplementary material for: MicroRNA-196a links human body fat distribution to adipose tissue extracellular matrix composition
Source: eBioMedicine. 2019 May 28;44:467–75. doi: 10.1016/j.ebiom.2019.05.047 (PMC6607082; doi:10.1016/j.ebiom.2019.05.047)
Supplement: Supplementary file 1 — Supplementary material [file mmc1.pdf]

SUPPLEMENTARY TABLES

Table S1: miRNAs with significantly different expression in abdominal subcutaneous and gluteal adipose tissue. Data represent ASAT biopsies from 15 men (8 lean and 7 overweight) and paired GSAT biopsies from 8 of the men (5 lean and 3 overweight) and indicate miRNAs that were significant with an uncorrected p-value of <0·05 and a minimum fold-change of 20%.

Table S2: Characteristics of the expanded panel of 40 individuals assembled for qPCR validation. Paired abdominal subcutaneous and gluteal adipose tissue biopsies were available for all participants. All participants were recruited from the Oxford Biobank.

Table S3: MicroRNAs differentially expressed in abdominal (ASAT) and gluteal (GSAT) adipose depots as determined by qPCR in the expanded panel of 40 individuals recruited from the Oxford Biobank (Table S1). p-values reaching the Bonferroni-adjusted significance threshold (p=0·00125) are indicated in bold.

Table S4: Associations between rs11614913 and body fat distribution in the meta-analysis of DXA data from Oxford Biobank, UK Biobank, EPIC-Norfolk and Fenland cohorts. For all conditions the rs11614913 T allele was the effect-increasing variant.

Figure S1: Figures A-F refer to expression in primary or immortalised *in vitro* differentiated pre-adipocytes derived from ASAT and GSAT (n=6; mean±SE; *p<0·05, **p<0·005, paired t-test). **A**. *HOXA5* in primary pre-adipocytes **B.** *HOXA5* in imAPAD and imGPAD cell lines **C**. *HOXB8* in primary pre-adipocytes **D**. *HOXB8* in imAPAD and imGPAD cell lines **E.** *HOXC8* in primary pre-adipocytes **F**. *HOXC8* in imAPAD and imGPAD cell lines.

Figure S2: We used summary-level GWAS data and a Bayesian model to generate credible sets of likely-causal SNPs in the HOXC locus. We performed this analysis in both the GIANT and UK Biobank (UKBB) data by first generating summary-level data for each of the independent signals discovered in the locus (rs10783615, blue points; rs1443512, yellow points; rs2071449, green points) using conditional analysis. After conditional analysis was complete, we computed the posterior probability for each SNP that it is the causal SNP in the region. This was repeated in the combined GWAS data (i.e., all samples), as well as in GWAS performed in sex-specific sample sets. rs11614913, location indicated by the dark grey triangle, has a modest posterior probability across all analyses, indicating that it is unlikely to be the causal variant for this particular GWAS signal at HOXC.

Table S1

MiRNA	Fold change (higher in gluteal)	p
**hsa-miR-196a**	2·00	$7{\cdot}9 \times 10^{-6}$
**hsa-miR-638**	1·93	$1{\cdot}4 \times 10^{-2}$
**hsa-miR-572**	1·85	$6{\cdot}5 \times 10^{-3}$
**hsa-miR-1225-5p**	1·69	$3{\cdot}2 \times 10^{-2}$
**hsa-miR-204**	1·67	$3{\cdot}5 \times 10^{-2}$
**hsa-miR-557**	1·64	$1{\cdot}9 \times 10^{-4}$
**hsa-miR-1228**	1·41	$5{\cdot}7 \times 10^{-3}$
**hsv1-miR-H1**	1·35	$3{\cdot}0 \times 10^{-2}$
**hsa-miR-188-5p**	1·34	$3{\cdot}8 \times 10^{-3}$
**hsa-miR-296-5p**	1·32	$9{\cdot}7 \times 10^{-4}$
**hsa-miR-1225-3p**	1·28	$6{\cdot}4 \times 10^{-3}$
**hsa-miR-140-3p**	1·27	$1{\cdot}2 \times 10^{-2}$
**kshv-miR-K12-10a**	1·27	$4{\cdot}3 \times 10^{-3}$
**hsa-miR-140-5p**	1·26	$4{\cdot}2 \times 10^{-2}$
**hsa-miR-550**	1·21	$8{\cdot}8 \times 10^{-3}$
**hsa-miR-1237**	1·20	$7{\cdot}4 \times 10^{-3}$
**hsa-miR-133a**	1·20	$4{\cdot}1 \times 10^{-4}$
**hsa-miR-18b***	1·20	$1{\cdot}8 \times 10^{-3}$
**hsa-miR-214***	0·82	$9{\cdot}0 \times 10^{-3}$
**hsa-miR-450a**	0·82	$1{\cdot}6 \times 10^{-3}$
**hsa-miR-424**	0·80	$4{\cdot}9 \times 10^{-2}$
**hsa-miR-10b***	0·79	$4{\cdot}0 \times 10^{-2}$
**hsa-let-7c**	0·78	$3{\cdot}1 \times 10^{-2}$
**hsa-miR-30a***	0·77	$4{\cdot}9 \times 10^{-2}$
**hsa-miR-199a-5p**	0·74	$8{\cdot}8 \times 10^{-3}$
**hsa-miR-199b-3p**	0·74	$2{\cdot}0 \times 10^{-2}$
**hsa-miR-125b**	0·73	$2{\cdot}9 \times 10^{-2}$
**hsa-miR-148a**	0·70	$4{\cdot}4 \times 10^{-2}$
**hsa-miR-10a**	0·68	$2{\cdot}0 \times 10^{-2}$
**hsa-miR-10b**	0·65	$6{\cdot}5 \times 10^{-3}$
**hsa-miR-335**	0·65	$4{\cdot}9 \times 10^{-2}$

Table S2

	Lean (BMI<25) Mean (SD)				Overweight (BMI>25) Mean (SD)				Lean vs overweight
	Men		Women		Men		Women		p-value
**N**	10		10		10		10		
**BMI (kg/m2)**	24·4	(0·5)	22·5	(1·4)	35·1	(6·3)	32·2	(3·7)	
**Waist circumference (cm)**	89·3	(5·2)	76·6	(4·0)	117·4	(14·0)	102·9	(9·9)	<0·001
**Hip circumference (cm)**	98·6	(2·4)	96·6	(4·5)	116·7	(10·3)	114·1	(8·2)	<0·001
**WHR**	0·9	(0·1)	0·8	(0·0)	1·0	(0·1)	0·9	(0·1)	<0·001
**Plasma glucose (mmol/l)**	5·3	(0·5)	4·8	(0·5)	5·5	(0·5)	5·3	(0·7)	0·15
**Plasma insulin (mU/l)**	8·7	(3·8)	8·2	(1·6)	21·8	(9·5)	12·6	(4·2)	<0·001
**Plasma Triglycerides (mmol/l)**	1·6	(1·5)	0·7	(0·2)	2·1	(0·9)	1·4	(0·4)	<0·001
**Plasma HDL cholesterol (mmol/l)**	1·2	(0·3)	1·5	(0·3)	1·0	(0·1)	1·2	(0·3)	0·013

Table S3

miRNA	Mean expression in ASAT (SEM)	Mean expression in GSAT (SEM)	Higher in ASAT or GSAT	Fold change	p-value	Fold change in men	p-value in men	Fold change in women	p-value in women
**miR-335**	0·73 (0·05)	0·58 (0·05)	ASAT	1·3	0·012	1·3	0·054	1·3	0·042
**miR-320**	0·51 (0·03)	0·42 (0·03)	ASAT	1·2	0·009	1·2	0·086	1·2	0·100
**miR-204**	0·26 (0·03)	0·60 (0·07)	GSAT	2·3	**$6{\cdot}2\text{x}10^{-8}$**	2·3	**0·001**	2·3	**$3{\cdot}8\text{x}10^{-5}$**
**miR-196a**	0·48 (0·04)	0·99 (0·06)	GSAT	2·3	**$9{\cdot}6\text{x}10^{-11}$**	2·2	**$2{\cdot}8\text{x}10^{-5}$**	1·9	**$2{\cdot}4\text{x}10^{-10}$**
**miR-27b**	0·73 (0·05)	0·97 (0·06)	GSAT	1·3	**$4{\cdot}1\text{x}10^{-5}$**	1·4	0·005	1·3	0·004
**miR-21**	0·46 (0·04)	0·61 (0·05)	GSAT	1·3	**0·001**	1·3	0·145	1·4	0·027
**miR-146b**	0·61 (0·09)	0·78 (0·11)	GSAT	1·3	0·002	1·2	0·28	1·4	0·038
**miR-196b**	0·80 (0·04)	1·19 (0·05)	GSAT	1·3	**0·001**	2·3	0·001	1·2	0·68
**miR-1225**	0·99 (0·07)	1·23 (0·09)	GSAT	1·2	0·002	1·5	0·006	1·2	0·22
**miR-146a**	0·41 (0·03)	0·47 (0·04)	GSAT	1·2	0·004	1·1	0·46	1·3	**0·001**
**miR-572**	1·58 (0·11)	1·93 (0·11)	GSAT	1·2	0·002	1·3	0·023	1·2	0·026
**miR-23b**	0·58 (0·03)	0·70 (0·04)	GSAT	1·2	0·008	1·4	0·006	1·1	0·21
**miR-221**	0·20 (0·03)	0·24 (0·03)	GSAT	1·2	0·042	1·1	0·44	1·3	**0·001**

Table S4

Measurement	N	β	Standard error	p-value
**Total android adipose tissue**	24010	0·018	0·0093	0·051
**Total gynoid adipose tissue**	24010	-0·0034	0·0093	0·71
**Visceral adipose tissue**	23945	-0·030	0·0093	0·0012
**Subcutaneous abdominal adipose tissue**	23945	0·053	0·0093	$1{\cdot}05 \times 10^{-8}$

.

Figure S1

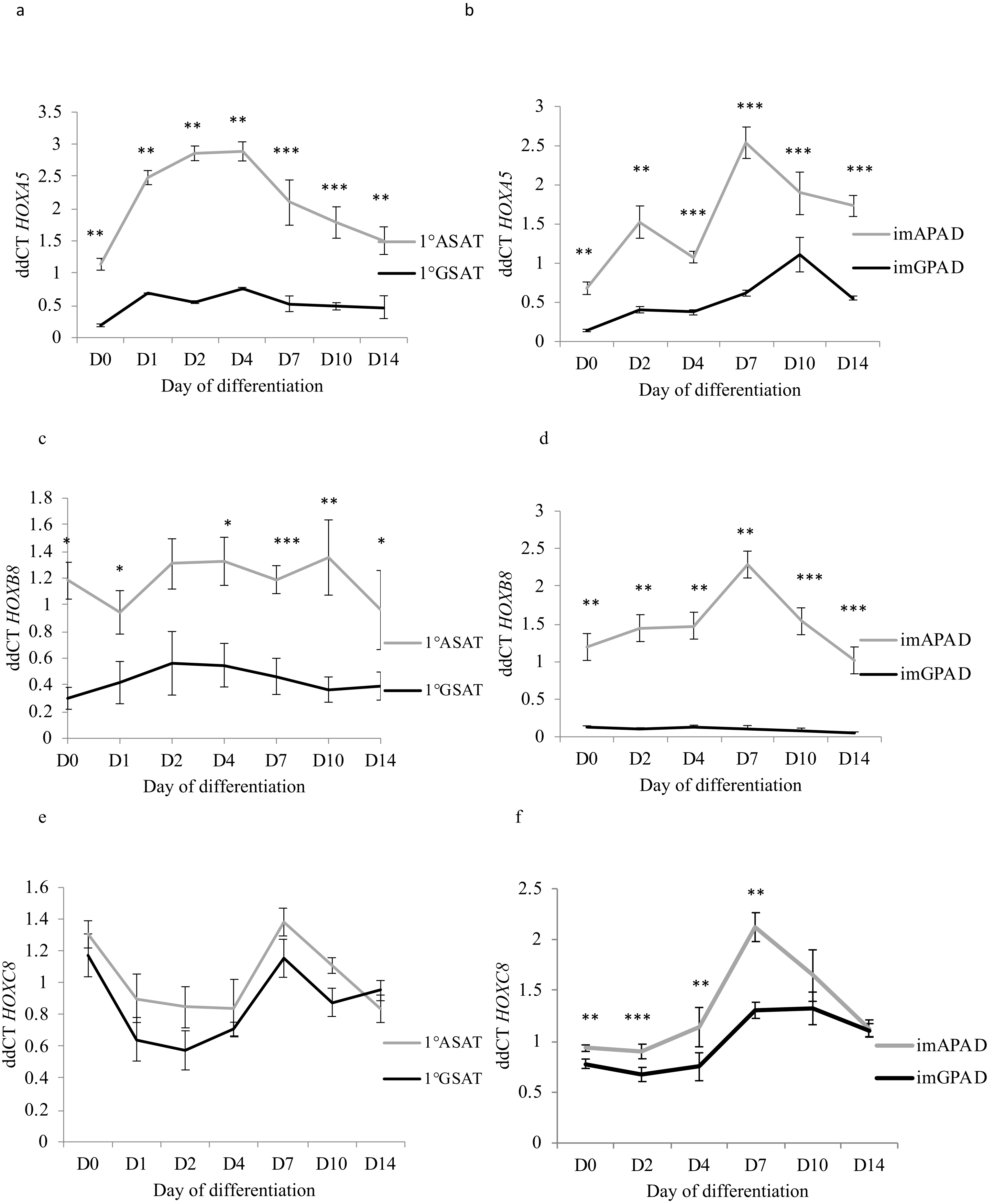
a
b
ddCT HOXA5
Day of differentiation
D0 D1 D2 D4 D7 D10 D14
1°ASAT
1°GSAT
D0 D2 D4 D7 D10 D14
imAPAD
imGPAD
c
d
ddCT HOXB8
e
f
ddCT HOXC8

Figure S2

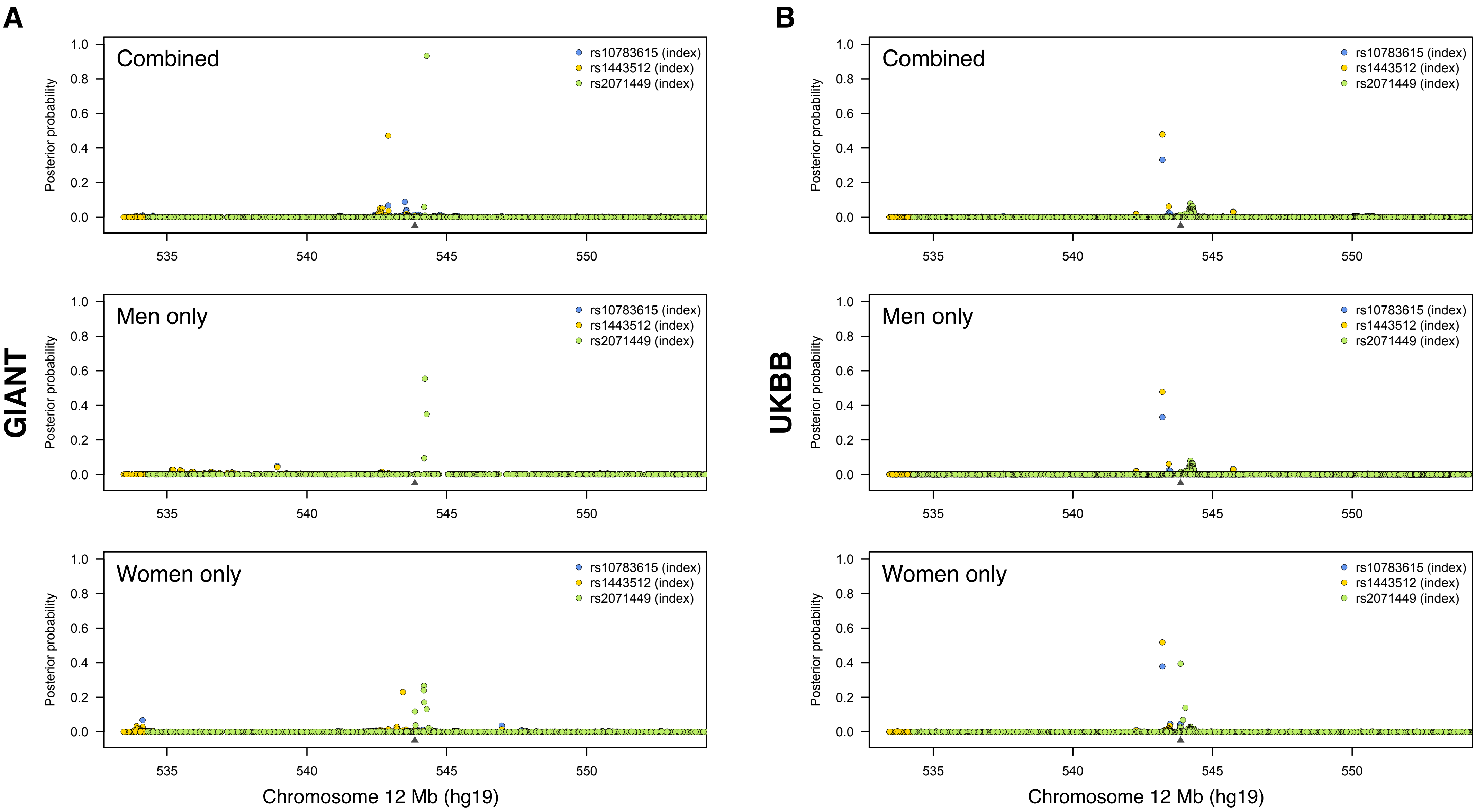
A
B
GIANT
UKBB
Combined
Men only
Women only
rs10783615 (index)
rs1443512 (index)
rs2071449 (index)
Posterior probability
1.0
0.8
0.6
0.4
0.2
0.0
535
540
545
550
Chromosome 12 Mb (hg19)
